# Hypophosphatasia Presenting as a Chronic Diffuse Pain Syndrome with Extra-Articular Calcifications

**DOI:** 10.3390/jcm13082263

**Published:** 2024-04-13

**Authors:** Florence Lehane, Olivier Malaise, Christian Von Frenckell, Bernard Otto, Elisa Docampo, Clio Ribbens

**Affiliations:** 1 Rheumatology Department, University Hospital of Liège, 4000 Liège, Belgiumclio.ribbens@chuliege.be (C.R.); 2 Radiology Department, University Hospital of Liège, 4000 Liège, Belgium

**Keywords:** hypophosphatasia, alkaline phosphatase, pain, calcium pyrophosphate deposition disease

## Abstract

Hypophosphatasia is a rare genetic disease characterized by abnormal alkaline phosphatase activity and deficiency of bone and teeth mineralization. Hypophosphatasia is well known in pediatrics with typical presentations in children, but mild forms can also be present in adults and are difficult to detect. We present the case of a 50-year-old woman referred for pain management, with a previous diagnosis of fibromyalgia. The association of clinical features (diffuse pain syndrome, early dental loosening, personal history of two fractures with osteoporosis, and family history of osteoporosis) with radiographic (heterotopic calcifications of the yellow and interspinous lumbar ligaments) and biological (low levels of total alkaline phosphatase) indices was suggestive of hypophosphatasia, which was confirmed by genetic analysis. We review and discuss the association between hypophosphatasia, musculoskeletal pain, and calcium pyrophosphate deposition and the importance of raising the diagnosis of adult-onset hypophosphatasia when facing these two rheumatologic entities.

## 1. Case Presentation

A 50-year-old pre-menopausal woman was referred to the rheumatology department for pain management. She suffered from diffuse pain involving small and large joints, but also the lumbar and cervical spine. The pain was mainly mechanical, without joint swelling. She also presented headaches, cognitive symptoms with intense fatigue, and Raynaud’s phenomenon (whitening of fingers in response to cold). These symptoms had been present for more than twenty years. Physical examination revealed no hand, elbow, foot, or knee synovitis. There was no limitation in the range of movement of peripheral joints, strength deficit, or loss of sensitivity. We observed axial stiffness and pain during active mobilization. Palpation of both joints and muscles elicited diffuse pain.

The personal medical history of the patient included a diagnosis ten years earlier of fibromyalgia (a chronic condition with widespread musculoskeletal pain, in the absence of any other etiology), a diagnosis two years earlier of osteoporosis (a bone densitometry demonstrated a total hip Z-score of −2.7, with shoulder and tibia fractures following a fall two years earlier, but no treatment had been initiated), as well as a dental prosthesis at the age of 22 because of early dental loosening. The family medical history highlighted osteoporosis in her father and her two sisters.

**Figure 1:**
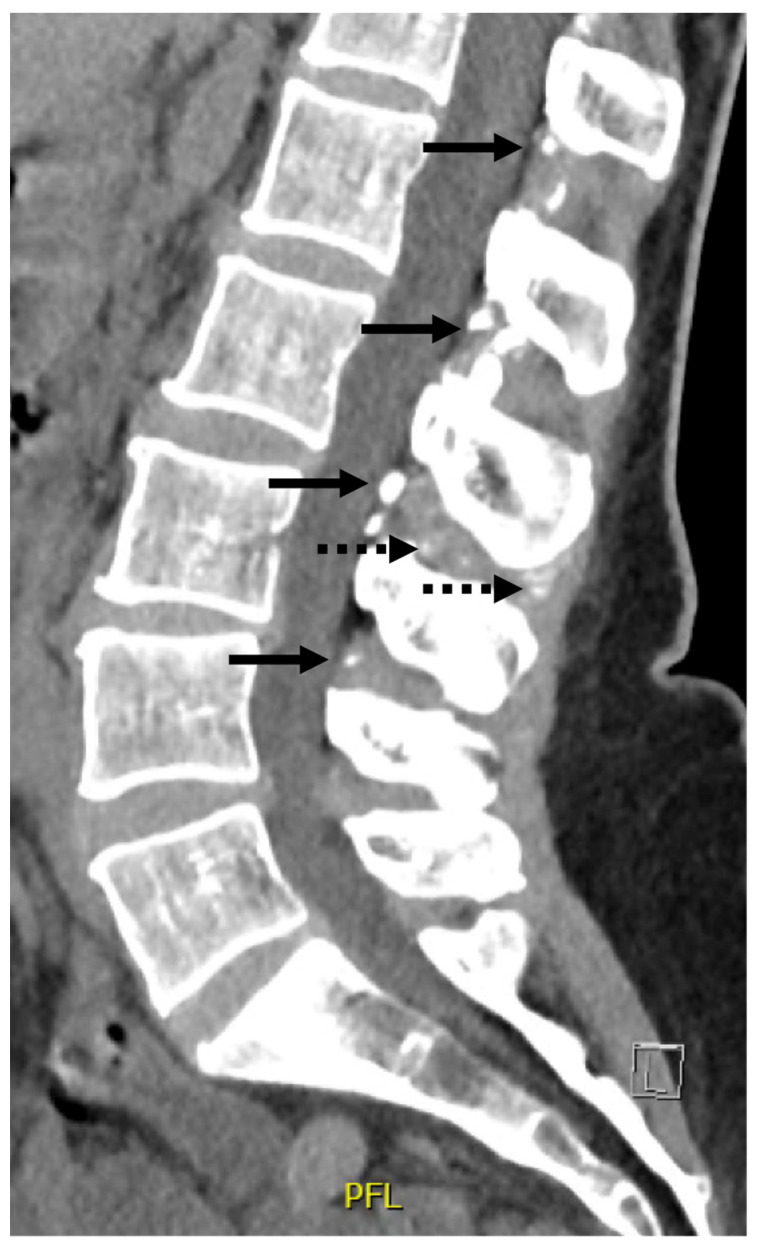
**Lumbar CT scan.** The lumbar CT scan, performed three months before the consultation, demonstrated diffuse degenerative disc disease with atypical heterotopic calcifications of the yellow ligament (arrows) and interspinous ligaments (dotted arrows).

Several medical imaging examinations had already been carried out in previous years. A whole-body bone scan, performed three years earlier, showed no argument in favor of an inflammatory arthropathy but did show signs of degenerative joint disease mainly affecting the shoulders and hands. Three months before the consultation, as lower back pain was getting worse, a lumbar CT scan was performed, which demonstrated diffuse degenerative disc disease with atypical heterotopic calcifications of the yellow ligament and interspinous ligaments, suggesting abnormal extra-articular calcium accumulation and calcium pyrophosphate deposition disease (Figure 1). Raynaud’s phenomenon had been evaluated several years ago by video-capillaroscopy, the results of which were normal.

Looking back at the previous blood tests available, recent blood evaluation demonstrated no inflammation, no markers of auto-immune disease (negative for antinuclear antibodies, rheumatoid factor, and anti-citrullinated antibodies), and normal glycemia, iron, and phospho-calcic parameters. However, we observed that total alkaline phosphatase levels were reduced (26 U/L, normal values: 40–150) and that these reduced values had already been observed ten years earlier (17 U/L, normal values: 40–150). The bone fraction of alkaline phosphatase was also below the normal range (2.3 microgram/L, normal values: 4.9–26.6).

Hypophosphatasia was therefore suspected. A genetic analysis was performed and found a pathogenic heterozygous variant (c.346G > A) of the ALPL gene (class 5 variant). The presence of a pathogenic variant of the ALPL gene (major criterion), chronic musculoskeletal pain, early atraumatic loss of teeth, and chondrocalcinosis (three minor criteria) allowed us to make a final diagnosis of hypophosphatasia [1]. Substrates of the enzyme tissue non-specific alkaline phosphatase enzyme (TNSALP) (pyridoxal phosphate, phosphoethanolamine, inorganic pyrophosphate) were not measured. This variant of the ALPL gene was also found in one of her sisters suffering from osteoporosis with low alkaline phosphatase levels but was not detected in the third sister suffering from osteoporosis but with normal alkaline phosphatase levels.

Colchicine was prescribed after imaging showed calcium pyrophosphate deposition disease, but it was not efficacious. The patient was referred to a chronic pain specialist. Despite osteoporosis, we did not introduce any anti-resorptive drug because of the diagnosis of hypophosphatasia.

## 2. Discussion

a. Definition of hypophosphatasia

Hypophosphatasia is an inborn error of metabolism caused by reduced or absent activity of the TNSALP enzyme, resulting from pathogenic variants of the ALPL gene [2]. Hypophosphatasia has a broad phenotypic spectrum, ranging from severe perinatal forms to milder adult ones. Up to now, more than 300 pathogenic variants associated with hypophosphatasia have been identified, and the huge variety in the type of pathogenic variant results in the highly variable expressivity of the disease. The main role of the TNSALP enzyme is the liberation of inorganic phosphate (Pi) for hydroxyapatite crystal propagation, leading to skeletal mineralization; a deficiency or absence of activity of this enzyme is responsible for abnormal mineralization of the growth plates, bones (osteomalacia), and teeth [2,3]. Low or reduced function of the ALPL gene also causes an increase in three phosphoric compounds: inorganic pyrophosphate (PPi), pyridoxal 5′phosphate (PLP), and phospho-ethanolamine (PEA), leading to the accumulation of these compounds that may be responsible for the neurological (pyridoxine-sensitive convulsions) and musculo-articular (microcrystalline arthropathy, fatigability/muscle hypotonia) signs [2].

b. Clinical presentation of hypophosphatasia

Depending on the age of onset, six clinical forms are defined: prenatal, perinatal, infantile, juvenile, adult, and odontohypophosphatasia [4]. Hypophosphatasia may be an autosomal dominant or autosomal recessive disease. The severe perinatal and the majority of infantile forms are inherited in an autosomal recessive mode [5]. In mild forms (benign prenatal, juvenile, adult, and odontohypophosphatasia), both the autosomal dominant and autosomal recessive modes of transmission have been identified [5]. In addition, the expressivity of the phenotype is variable and penetrance is incomplete. This range of inheritance patterns partially explains the clinical heterogeneity. Perinatal- and infantile-onset hypophosphatasia are often severe and can be lethal without treatment. In contrast, mild forms of hypophosphatasia can be present in adults and are difficult to detect. Adult hypophosphatasia typically presents during middle age, around the age of 40. It can present under multiple facets (fractures and musculoskeletal pain are commonly observed, but also delayed bone healing, osteomalacia, arthropathy, altered gait, or early loss of teeth) but can also be asymptomatic [2,3,4].

c. Musculoskeletal pain and hypophosphatasia

Musculoskeletal pain is a common complaint in adult-onset hypophosphatasia. In a Spanish population with low alkaline phosphatase, half of the subjects suffered from mild skeletal or muscular pain [6]. Musculoskeletal pain was also described in 41% of patients with hypophosphatasia in a retrospective cohort of the Mayo Clinic [7]. Nevertheless, these studies have to be interpreted with caution since, in most of the cases, genetical testing was not available and other causes could explain low ALP levels such as hypothyroidism, malnutrition, magnesium deficiency, and glucocorticoid or anti-resorptive treatments. Schmidt et al. presented a cohort of 38 patients with hypophosphatasia (with a genetic confirmation for 32 of them), in whom muscular and joint pain were observed in 61 and 38% of the cases, respectively [8]. In a survey conducted in patients with established hypophosphatasia, the rate of reported pain was as high as 96%, mainly affecting bones and joints (82 and 73%, respectively) but also muscle (53%) [9]. In total, 62% of them reported daily use of analgesic drugs. In another cohort, all the patients included declared musculoskeletal pain [10]. Moreover, in a cohort of patients with persistent biological hypophosphatasia, those with a genetic variant of the ALPL gene reported statistically more musculoskeletal pain than those with negative genetic tests [11]. However, a second similar study among patients with persistent low alkaline phosphatase showed no difference in terms of pain and quality of life between patients with or without ALPL mutations, but these two subgroups were significantly different from the healthy controls in terms of pain [12]. It should be underlined that the quality of life of patients with hypophosphatasia is significantly lower than in control patients. In 35 patients with hypophosphatasia, the authors demonstrated that body pain and pain perception were higher in the patient group than in controls [12]. In addition, patient scores were worse than controls in several items of the SF-36 questionnaire, which investigates health-related quality of life (physical, body pain, emotional roles) [12]. This should be kept in mind when facing a patient with hypophosphatasia. Medical care should not be limited to fracture prevention, dental care, or pain treatment, but it should also include psychological help if needed.

d. Fibromyalgia and hypophosphatasia

Hypophosphatasia can be confused with fibromyalgia. Fibromyalgia is characterized by chronic widespread pain and other symptoms (such as stiffness, sleeping disorders, chronic fatigue, cognitive dysfunction, memory disorders, and depressive episodes) in the absence of other diseases that could explain the pain [13]. The absence of specific diagnostic criteria opens the door to misdiagnosis, if “the other cause that could explain the pain” is not discovered. In a study including nineteen patients presenting low ALP levels not explained by secondary causes, three had previously been misdiagnosed as having chronic fatigue syndrome and/or fibromyalgia [14]. In the series published by Hogler et al., 9.5% of the 148 adults with hypophosphatasia had a previous diagnosis of fibromyalgia [15]. Lastly, a retrospective chart review of 305 patients diagnosed with fibromyalgia showed that up to 19% of fibromyalgia patients aged 18 years or older were found to have consistently low levels of alkaline phosphatase and suspicion for undiagnosed underlying hypophosphatasia (but the authors also noted that 28% of the patients with low alkaline phosphatase were taking bisphosphonates, suggesting that all patients with low alkaline phosphatase probably did not suffer from hypophosphatasia) [16].

The etiology of hypophosphatasia-associated pain remains uncertain and is probably multifactorial. Several hypotheses can be raised. First, pain in long bones can be related to osteomalacia, as observed in metabolic bone disease (but it is interesting to note that musculoskeletal symptoms and pain are independent of skeletal involvement in the Global HPP Registry [17]). Second, ALPL is expressed in brain neuronal cells and muscle cells during development and adulthood, and it is also associated with the synthesis of neurotransmitters in the central nervous system, contributing to neuromuscular and pain manifestations [18]. Third, we must keep in mind that chronic pain is a frequent symptom in the adult population and chronic pain can co-exist with hypophosphatasia. Lastly, pain in hypophosphatasia can also be due to microcrystalline disease.

e. Calcium pyrophosphate crystal deposition and hypophosphatasia

Calcium pyrophosphate crystal deposition in and around joints is often observed in hypophosphatasia. A large retrospective study identified US adult patients with persistent hypophosphatasemia and showed more frequent radiographic calcific periarthritis, diffuse idiopathic skeletal hyperostosis (DISH), and chondrocalcinosis (*p* < 0.001) [19]. In another case series of 29 patients with low ALP identified in a rheumatology unit, Feurstein et al. demonstrated that patients with ALPL pathogenic variants/mutations have significantly more peri-articular calcifications (but also dental problems) [20]. Out of 22 adults diagnosed with hypophosphatasia, the Mayo Clinic identified radiographic calcium pyrophosphate deposition disease in 27% and documented pyrophosphate arthropathy in 14% [7], while 6 out of 16 patients had symptomatic chondrocalcinosis [21] and 21% had crystal disease in another cohort [8]. A recent study described a case series of 14 patients presenting to rheumatology, with a final diagnosis of hypophosphatasia [22]. The most common presentation was peripheral joint pain (in 86%), which was mainly due to early-onset calcium pyrophosphate deposition disease (in 71%), but also osteoarthritis in 50% and bursitis in 50%. Axial pain was also reported in 64% due to osteoarthritis or spinal stenosis. Calcium pyrophosphate deposition disease prevalence was higher in this study; it could be the cause of a referral bias: patients presenting with inflammatory joint pain present more often to rheumatology and calcium pyrophosphate deposition disease is not easily diagnosed by other physicians. The authors hypothesize that the high rate of osteoarthritis and bursitis was secondary to calcium pyrophosphate deposition disease. Pyrophosphate deposition in hypophosphatasia can be explained: in physiological conditions, ALPL converts inorganic pyrophosphate (PPi) to inorganic phosphate (Pi), which can complex with ionized calcium and constitute hydroxyapatite crystals, required for correct mineralization. ALPL deficiency leads to the accumulation and an excess of inorganic pyrophosphate (PPi), but also of calcium, which can complex and accumulate.

f. Diagnostic criteria of hypophosphatasia

The HPP International Working Group recently carried out a comprehensive review of recent published literature, with the aim of creating specific diagnostic guidelines for HPP throughout the lifespan, given the heterogeneous presentation of hypophosphatasia in adults [1]. This led to the definition of major and minor diagnostic criteria. Major diagnostic criteria include ALPL gene variants, elevation of natural substrates of TNSALP, atypical femur fractures, and recurrent metatarsal stress fractures. Minor diagnostic criteria include poorly healing fractures, chronic musculoskeletal pain, early atraumatic loss of teeth, chondrocalcinosis, and nephrocalcinosis. The working group suggests that if an adult patient meets two major criteria, or one major criterion and two minor criteria, he/she meets the criteria for the clinical diagnosis of hypophosphatasia. Among musculoskeletal or rheumatological-like manifestations that can be encountered in hypophosphatasia, this literature review highlighted myopathy, chronic muscle pain with or without arthropathy, muscle weakness, joint pain, enthesopathy, bone pain, altered gait, impaired mobility, and calcium pyrophosphate deposition disease. Overall, the pooled prevalence of chronic musculoskeletal pain and chondrocalcinosis/joint CPPD was 64% and 16%, respectively.

Enzyme replacement therapy (asfostase alfa) is approved to treat pediatric-onset hypophosphatasia. In adults, treatment is usually only supportive. However, asfostase is sometimes used. The global HPP Registry collected data from 190 adult patients receiving asfostase [23]. They interestingly demonstrated that adult patients with pain related to hypophosphatasia could also benefit from this treatment: adults who received asfostase alfa for 6 months (or more) experienced significant improvements in mobility, physical function, and health-related quality-of-life indices, which were maintained over 3 years of follow-up. Supportive treatment in adults was reviewed by Seefred et al. [24]. It included dental care and oral hygiene, physical therapy, and the use of pain-killers. Regarding pain, the co-existence of crystal deposition disease should be kept in mind, as it opens the door to additional treatment (colchicine, joint injection, etc.). As previously discussed, quality of life is significantly reduced among patients with hypophosphatasia and psychological help should be proposed to these patients. Lastly, bone disease prevention is important, keeping in mind that anti-resorptive drugs should be avoided. In view of all these therapeutic modalities, multidisciplinary care is necessary to offer a holistic treatment.

Coming back to our clinical case, some points should be kept in mind. This case illustrates the fact that hypophosphatasia is not well known among physicians, as our patient had low values of total alkaline phosphatase for at least ten years before the diagnosis. None of the clinical signs of hypophosphatasia that our patient presented (early loss of teeth, chronic pain syndrome, chondrocalcinosis) had been recognized over many years nor put in relation to the low alkaline phosphatase levels. In addition, hypophosphatasia was not even thought of after a bone fracture and an additional diagnosis of osteoporosis (while active screening for hypophosphatasia is now a recommendation during osteoporosis work-up).

## 3. Conclusions

Adult-onset hypophosphatasia has no specific symptoms or clinical signs. If the diagnosis is suspected when facing atypical fractures or early bone loss, musculoskeletal pain and abnormal calcifications are two of the main symptoms of this disease in adults. Hypophosphatasia should be suspected, and alkaline phosphatase reduction must be sought, when patients complain of diffuse pain or when a diagnosis of calcium pyrophosphate deposition disease is established.

Adult-onset hypophosphatasia should be differentiated from fibromyalgia to provide the patients with an explanation for their long-experienced pain. The use of anti-osteoporotic drugs that inhibit bone resorption (bisphosphonates, denosumab, etc.) should be avoided in hypophosphatasia and international recommendations therefore propose alkaline phosphatase level evaluation before any anti-resorptive drug prescription [25]. The frequent co-existence of chondrocalcinosis provides therapeutic options with colchicine or local joint injection. Finally, we can hope that enzyme replacement therapy will also find a place in the therapeutic strategies for hypophosphatasia in adults.
